# Towards a Universal SMILES representation - A standard method to generate canonical SMILES based on the InChI

**DOI:** 10.1186/1758-2946-4-22

**Published:** 2012-09-18

**Authors:** Noel M O’Boyle

**Affiliations:** 1 Analytical and Biological Chemistry Research Facility, Cavanagh Pharmacy Building, University College Cork, Cork, Co. Cork, Ireland

**Keywords:** Line notations, InChI, SMILES, Canonicalisation

## Abstract

**Background:**

There are two line notations of chemical structures that have established themselves in the field: the SMILES string and the InChI string. The InChI aims to provide a unique, or canonical, identifier for chemical structures, while SMILES strings are widely used for storage and interchange of chemical structures, but no standard exists to generate a canonical SMILES string.

**Results:**

I describe how to use the InChI canonicalisation to derive a canonical SMILES string in a straightforward way, either incorporating the InChI normalisations (Inchified SMILES) or not (Universal SMILES). This is the first description of a method to generate canonical SMILES that takes stereochemistry into account. When tested on the 1.1 m compounds in the ChEMBL database, and a 1 m compound subset of the PubChem Substance database, no canonicalisation failures were found with Inchified SMILES. Using Universal SMILES, 99.79% of the ChEMBL database was canonicalised successfully and 99.77% of the PubChem subset.

**Conclusions:**

The InChI canonicalisation algorithm can successfully be used as the basis for a common standard for canonical SMILES. While challenges remain – such as the development of a standard aromatic model for SMILES – the ability to create the same SMILES using different toolkits will mean that for the first time it will be possible to easily compare the chemical models used by different toolkits.

## Background

Line notations are linear representations of chemical structures that encode the connection table and (usually) the stereochemistry of a molecule as a line of text [1]. They are widely used for storing, representing, communicating and checking the identity of chemical structures. Their popularity derives from one or more of the following: they encode the chemical structure in a compact form; they may be human-readable and/or human-writable; they are easily entered into software (for example, by copying and pasting into a text entry box on a website or a dialog box in a GUI, or entered into a spreadsheet cell); they may be canonical (that is, provide a unique representation for a particular molecule), in which case they may easily be used to check identity, search databases or even search the web.

While line notations typically do not allow the incorporation of additional information beyond the connection table and its associated chemistry (with the notable exception of SYBYL Line Notation [2,3]), even where the underlying data is stored in a 2D or 3D file format as in several web databases (for example, PubChem [4]), linear representations of the data are usually provided for convenience. Apart from IUPAC nomenclature [5], the two most widely used line notations and the focus of the current work are the SMILES (Simplified Molecular Input Line Entry System) string developed by Weininger [6] and Daylight Chemical Information Systems [7], and IUPAC’s InChI (International Chemistry Identifier) representation [8,9]. Others include SLN (SYBYL Line Notation), ROSDAL [10] (from the Beilstein Institute), WLN (Wiswesser Line Notation [11]), MCDL (Modular Chemical Descriptor Language [12,13]), the InChIKey (a hashed representation of the InChI) and more [1,14-20].

The SMILES format is the most popular line notation in use today. Created by David Weininger in 1986 at the US Environmental Research Laboratory (USEPA), and further developed at the company he co-founded, Daylight Chemical Information Systems, the SMILES format is particularly attractive as it is easily learnt, is both human-readable and -writable, and encodes stereochemistry in an intuitive way. Since no formal specification of the SMILES format was ever published and there are several ambiguities that have led to differences in implementation, in 2007 Craig James (eMolecules, Inc., and formerly of Daylight) initiated a community approach to develop a specification for SMILES, the OpenSMILES specification [21]. The SMILES format is not without some drawbacks: it is focused on molecules whose bonds fit the 2-electron valence model, it handles a limited array of stereochemistry types, and as yet there is no standard for handling aromaticity. However, perhaps the greatest limitation of the SMILES format is that there is no standard way to generate a canonical representation. While Weininger et al. [22] did publish a canonicalisation procedure (CANGEN) for SMILES, the procedure did not include a treatment of stereochemistry, one of the most difficult aspects of the problem. Daylight subsequently provided a commercial product to generate canonical SMILES but as the algorithm was proprietary, other commercial and open-source software developed their own algorithms for generating canonical SMILES all of which differed from each other and none of which are published.

In 1999, the need for a community standard for a canonical linear representation led to a proposal by Steve Heller and Steve Stein at the National Institute of Standards and Technology (NIST) in the US for a new representation, the InChI (International Chemical Identifier), which was subsequently developed as an IUPAC initiative in collaboration with NIST [8]. The first version of the InChI was released in 2005, and in 2009 the InChI Trust [9] was formed to oversee its development. The goal of the InChI is to provide an canonical representation that can be used to link information from different databases on the same molecules. To do this, the InChI algorithm combines a normalisation procedure, a canonicalisation algorithm, and a layered structure that helps identify isomers.

This work describes a method to generate canonical SMILES using canonical labels from the InChI. While other canonicalisation methods have been developed that take stereochemistry into account (for example, Koichi et al. [16] as well as all of the (unpublished) methods used by the various cheminformatics toolkits), only the InChI is suitable for the development of a standard canonical SMILES string that can easily be supported by many different software libraries, as there exists only a single implementation, the code for which is freely available under an Open Source license. This implementation has been incorporated into several Open Source cheminformatics libraries (Open Babel [23], the Chemistry Development Kit [24], RDKit [25], Chemkit [26] and Indigo [27]) as well as proprietary software from several companies (for example, ACD/ChemSketch [28], CACTVS [29], JChem [30], and planned for OEChem [31]). This means that all of these programs can generate the same InChI as the official InChI software, and thus they have the capability to generate the same canonical SMILES.

Here I describe Universal SMILES and Inchified SMILES, easily-implemented methods that use the canonical labels provided by the InChI to generate canonical SMILES. The term Universal is used as the method can be universally adopted by any software with access to the InChI library or executable, without the need for any changes to the InChI software. These SMILES strings do not use any extensions to the SMILES standard, and so are completely interchangeable with the existing SMILES strings used by many databases. The advantage of replacing existing SMILES strings with Universal SMILES or Inchified SMILES is that the ease of use and readability of SMILES strings is enhanced by the indexing and linking ability associated with a canonical representation such as the InChI.

This is the first description of a method to generate canonical SMILES that takes stereochemistry into account. Apart from yaInChI (a modification of the InChI by Cho et al. [32]), it is also the first time that the canonical labels from the InChI have been used to generate an alternative canonical representation, although in fact the idea itself has previously been proposed by Murray-Rust (on the Open Babel mailing list, February 2005 [33]). However, there are other studies that share the idea of exploiting information contained in the InChI for purposes other than uniquely identifying a molecule. Thalheim et al. [34] implemented a tautomer enumeration procedure based on information contained in the InChI. The InChI normalises all (supported) tautomers to the same representation, and stores the normalised information in the mobile hydrogen layer of the InChI which describes how one or more hydrogens is shared between a set of heteroatoms. The authors extracted this layer, and developed an algorithm that generated all of the tautomers consistent with it. The layered structure of the InChI can be exploited to identify isomers of various types; in a crystallographic study, Fábián and Brock [35] used the enantiomer layer to identify a particular class of racemic crystal, kryptoracemates, where the enantiomers are not related by space-group symmetry.

I propose two approaches for the generation of a canonical SMILES based on the InChI, one of which includes the normalisation steps introduced by the InChI:

(1) The **Inchified SMILES** can be considered as a canonical SMILES string that corresponds to the Standard InChI. All of the normalisations applied to the structure by the InChI are passed onto the Inchified SMILES, and there is a one-to-one relationship between the two.

(2) In contrast, the **Universal SMILES** retains the original structure (and tautomeric state) but uses the canonical labels from the InChI to create a canonical SMILES string. It can be considered a drop-in replacement for existing SMILES, with the added benefit of being a canonical representation.

The Methods section describes how to generate Inchified and Universal SMILES. The Results section covers how these approaches were tested by implementing them as part of the Open Babel cheminformatics toolkit. Additional comments on the implementation as well as the implications of a widely-available standard canonical form for SMILES are contained in the Discussion.

## Methods

### Overview

**Figure 1:**
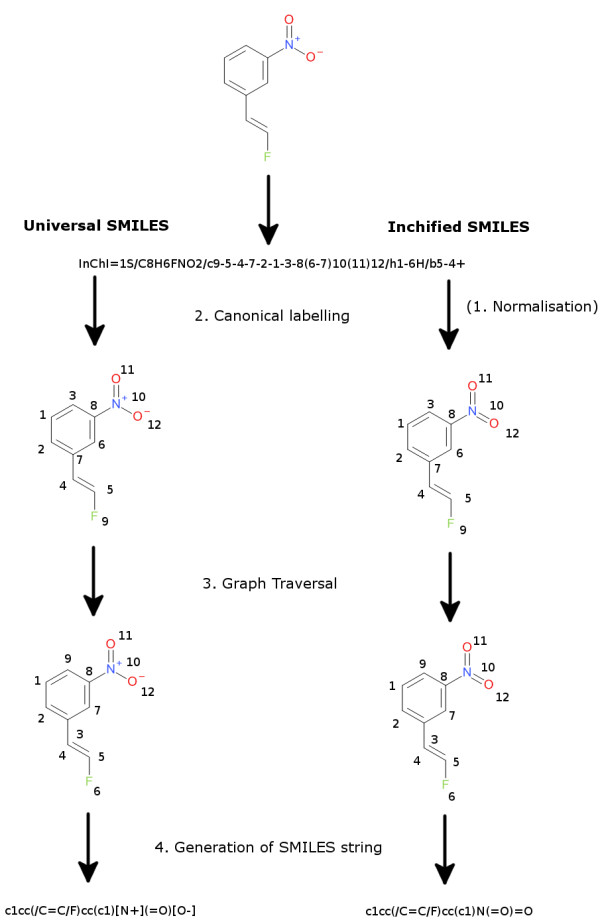
**An overview of the steps involved in generating Universal and Inchified SMILES.** The normalisation step just applies to Inchified SMILES. To simplify the diagram a Standard InChI is shown, but in practice a non-standard InChI (options *FixedH* and *RecMet*) is used for Universal SMILES.

The generation of an Inchified SMILES string involves four overall steps: structure normalisation, canonical labelling, tree traversal, and SMILES generation (Figure 1). The procedure for generating Universal SMILES does not include the normalisation step, and incorporates information from the InChI in a different way:

(1) **Inchified SMILES:** Generate a Standard InChI for a molecule *mol1* and use the InChI library to convert it back to a molecule *mol2*. The atoms of *mol2* will be in a canonical order although its connection table may be different to *mol1* due to normalisation. Using the canonical order, generate a canonical SMILES for *mol2*.

(2) **Universal SMILES:** Generate a non-standard InChI for a molecule *mol1*, and parse the auxiliary information to obtain the canonical labels. Using the canonical labels, generate a canonical SMILES for *mol1*.

In the absence of any normalisations affecting the Inchified SMILES, or differences between the Standard and non-standard InChIs, the Universal SMILES and Inchified SMILES for a particular molecule will be identical.

### Structure normalisation

When generating a Standard InChI a set of normalisations are first applied to the supplied structure. The purpose of these normalisations is to make sure that different ways of drawing the same structure will all yield the same InChI. For example, tautomers that differ by (1,3)-shifts of H atoms between heteroatoms may be normalised to the same representation; ion-pair representations (e.g. nitro group as –[N+]([O-])=O) are replaced by expanded valence representations (e.g. nitro group as –N(=O)=O). Other more complex normalisation steps are also applied to handle radicals and movable charges.

The Inchified SMILES incorporates these normalisation steps in a rather straightforward way. An InChI is generated from the original structure, and then that InChI is converted back to a molecular structure using the InChI library. Several structures with different connection tables may give the same InChI due to normalisation, but that InChI will only be converted back to a single structure, the normalised structure. For Inchified SMILES, the original structure is discarded and the normalised structure is used instead.

It is worth emphasising that the normalisations incorporated by the Inchified SMILES are completely dependent on the Standard InChI. If the Standard InChI does not detect and normalise different tautomeric states of a molecule (which happens for various classes of tautomers), different tautomers will not yield the same Inchified SMILES.

### Canonical labelling

In order to generate a unique, or canonical, representation for a molecule, one needs a way to canonically label the atoms of the molecule. Such a method will always give the same label to the same atom no matter how the atoms of the molecule are presented. As discussed above, different canonicalisation procedures have been developed by a variety of software vendors and open source projects. Here the canonical labels provided by the InChI canonicalisation algorithm are used.

Although a full and detailed description of the InChI canonicalisation algorithm has not been published, the InChI Technical Manual [36] presents an overview and Tchekhovskoi (of the InChI development team) has given some details in emails to the InChI public mailing list (see Apodaca [37]). The hydrogenless molecular graph is first canonicalised, based on an initial partitioning of the atoms similar to that described by Weininger for SMILES. Canonical numbering is then obtained using a method described by Agarwal and Gelernter [38], but using McKay’s Nauty algorithm [39] to improve efficiency. Further canonicalisation steps are then carried out in a stepwise manner for each additional InChI layer, while keeping previous layers fixed.

When generating Inchified SMILES, the preceding structure normalisation step returns the atoms of the molecule in InChI canonical order, so no further work is required. To generate Universal SMILES, the canonical order must instead be obtained by generating an InChI for the molecule and extracting the order from the InChI auxiliary information.

The relationship between the input order and the InChI canonical labels is given by the /N layer in the auxiliary information (output by default if using the InChI command-line application, or requested with *–xa* if using Open Babel). For example, for the input SMILES string ClCC(=O)Br (see Table 1) the /N layer indicates that canonical labels 1 through 5 correspond to input atoms 2, 3, 5, 1 and 4 respectively (e.g. canonical label 3 is input atom 5, the Br). Disconnected structures (such as C.C in Table 1) are handled similarly but with a semicolon separating the labels for each structure. Note that only bridging hydrogens are given canonical labels; neither hydrogens in general nor specific hydrogen isotopes receive them (see C([H])C and C([2H])O in Table 1).

**Table 1:** Universal SMILES for structures discussed in the text showing the input SMILES, and the corresponding non-standard InChI and auxiliary information

**SMILES**	Non-standard InChI*	**InChI Auxiliary info**	**Universal SMILES**
OCC	InChI=1/C2H6O/c1-2-3/h3H,2H2,1H3	AuxInfo=1/0/N:3,2,1/rA:3OCC/rB:s1;s2;/rC:;;;	CCO
ClCC(=O)Br	InChI=1/C2H2BrClO/c3-2(5)1-4/h1H2	AuxInfo=1/0/N:2,3,5,1,4/rA:5ClCCOBr/rB:s1;s2;d3;s3;/rC:;;;;;	C(C(=O)Br)Cl
C.C	InChI=1/2CH4/h2*1H4	AuxInfo=1/0/N:1;2/rA:2CC/rB:;/rC:;;	C.C
C.CC	InChI=1/C2H6.CH4/c1-2;/h1-2H3;1H4	AuxInfo=1/0/N:2,3;1/E:(1,2);/rA:3CCC/rB:;s2;/rC:;;;	CC.C
C([H])C	InChI=1/C2H6/c1-2/h1-2H3	AuxInfo=1/0/N:1,3/E:(1,2)/rA:3CHC/rB:s1;s1;/rC:;;;	CC
C([2H])O	InChI=1/CH4O/c1-2/h2H,1H3/i1D	AuxInfo=1/0/N:1,3/rA:3CH.i2O/rB:s1;s1;/rC:;;;	C([2H])O

* InChI generated using the non-standard options *RecMet* and *FixedH.*

For the purpose of generating Universal SMILES, two non-standard InChI options are used: *FixedH* (include fixed hydrogen layer) and *RecMet* (reconnect disconnected metals). The former allows us to correctly relate the input order and the canonical labels in certain cases where the molecular symmetry is broken only by a protonation state. For example, without this option, the following two SMILES strings for the same molecule, C(=O)([O-])C(=O)O and C(=O)(O)C(=O)[O-], give different Universal SMILES as the corresponding atoms receive different canonical labels in each case (see Table 2). Once *FixedH* is used, a fixed hydrogen layer (/f) is added to the InChI when mobile hydrogens are present, and the auxiliary information contains a corresponding /F section describing the correspondence between the input atom order and the canonical labels. Once this is used the same Universal SMILES can be generated in each case (Table 2).

**Table 2:** The effect of using the non-standard option FixedH on the InChI and Universal SMILES

			
**SMILES**	**Standard InChI**	**InChI Auxiliary info**	**“Universal” SMILES**
C(=O)([O-])C(=O)O	InChI=1/C2H2O4/c3-1(4)2(5)6/h(H,3,4)(H,5,6)/p-1	AuxInfo =1/1/**N:1,4,2,3,5,6**/E:(1,2)(3,4,5,6)/gE:(1,2)/rA:6COO-COO/rB:d1;s1;s1;d4;s4;/rC:;;;;;;	C(=O)(C(=O)O)[O-]
C(=O)(O)C(=O)[O-]	InChI=1/C2H2O4/c3-1(4)2(5)6/h(H,3,4)(H,5,6)/p-1	AuxInfo=1/1/**N:1,4,2,3,5,6**/E:(1,2)(3,4,5,6)/gE:(1,2)/rA:6COOCOO-/rB:d1;s1;s1;d4;s4;/rC:;;;;;;	C(=O)(C(=O)[O-])O
**SMILES**	Non-standard InChI*	**InChI Auxiliary info**	**Universal SMILES**
C(=O)([O-])C(=O)O	InChI=1/C2H2O4/c3-1(4)2(5)6/h(H,3,4)(H,5,6)/p-1/fC2HO4/h3H/q-1	AuxInfo=1/1/N:1,4,2,3,5,6/E:(1,2)(3,4,5,6)/gE:(1,2)/**F:4,1,6,5,2,3**/E:(5,6)/rA:6COO-COO/rB:d1;s1;s1;d4;s4;/rC:;;;;;;	C(=O)(C(=O)[O-])O
C(=O)(O)C(=O)[O-]	InChI=1/C2H2O4/c3-1(4)2(5)6/h(H,3,4)(H,5,6)/p-1/fC2HO4/h3H/q-1	AuxInfo=1/1/N:1,4,2,3,5,6/E:(1,2)(3,4,5,6)/gE:(1,2)/**F:1,4,3,2,5,6**/E:(5,6)/rA:6COOCOO-/rB:d1;s1;s1;d4;s4;/rC:;;;;;;	C(=O)(C(=O)[O-])O

When generating a Standard InChI, ligands are disconnected from any metal atoms present in order to normalise different representations of the same metal-ligand bonding system. This can cause some problems when trying to relate the input atoms and the canonical labels. For example, if you consider the platinum complex represented by the SMILES string C(=O)O[Pt](N)(N)Cl, when disconnected the two oxygens of the carboxy are considered equivalent by the InChI and so the canonical labels for the oxygens in the input structure may interchange depending on their atom order. To avoid such problems, the *RecMet* option is used when generating the InChI. This adds a reconnected metal layer (/r) to the InChI if a metal atom has been disconnected, and a corresponding /R section to the auxiliary information. The /R section has its own /N section (and its own /F section as described above if mobile hydrogens are present) which contains the correspondence between the input atom order and the canonical labels.

The following rule describes from which section the canonical labels should be extracted:

Rule A: The correspondence between the input atom order and the InChI canonical labels should be obtained from the reconnected metal layer (/R:) in preference to the initial layer, and then from the fixed hydrogen labels (/F:) in preference to the standard labels (/N:).

See the Additional file 1 for a Python script that extracts the canonical labels from an InChI as described by Rule A.

Here only the fixed hydrogen and reconnected metal layers have been considered. Another layer that may be present is the isotopic layer, and there may be an updated canonical label section associated with this. As of writing, there is a known bug in the InChI output for this section [40], and support for this additional layer will be added once this is addressed.

### Graph traversal

A SMILES string is a linear representation of a molecule created from a traversal of the molecular graph. The most straightforward way to traverse the graph is using a depth-first search (this minimises bond closure symbols). As discussed by Weininger et al. [22] the only questions that remain are where to start the traversal, and which branch to follow at each branch point:

Rule B: Start the graph traversal at the atom with the lowest canonical label. For disconnected structures, visit each structure in order of its lowest canonical label. (Modified by Rule E)

Rule C: At each branch point, multiple bonds are favoured over single or aromatic bonds, and lower canonical labels over higher. (Modified by Rule D)

Rule C follows Weininger et al. and reduces the probability of bond closures involving multiple bonds.

#### Handling hydrogens

In general, hydrogens are neither given a canonical label by the InChI nor included in the connection layer. However, for certain specific cases involving species consisting wholly of hydrogen atoms or with bridging hydrogens, one or more hydrogens may be labelled. For its part, in general a Universal or Inchified SMILES string does not include hydrogens (or rather they are included implicitly) as prescribed by Rule F below. However explicit hydrogens are used when indicating isotopes of hydrogen (i.e. [2H], [3H]), hydrogens attached to tetrahedral stereocentres with defined stereochemistry, and for the special cases of dihydrogen and hydrogen ions ([H][H], [H-], [H+]).

For explicit hydrogen atoms that are labelled by the InChI, Rules B and C should be followed. The following rule describes how to handle unlabelled explicit hydrogens:

Rule D: An explicit hydrogen atom in the SMILES string which is unlabelled by the InChI should be visited prior to other singly-bonded branches of the preceding atom (and favouring deuterium first over tritium), or if present at a tetrahedral stereocentre with defined stereochemistry, it should be written inside the square brackets (that is, [C@H] rather than [C@]([H])).

Example:

C([2H])([3H])Cl rather than C(Cl)([3H])[2H]

In practice, the first clause of Rule D can be ensured by giving such hydrogens a low canonical number so that Rule B will work without modification.

#### Handling groups whose order is not canonical

As a consequence of its normalisation rules, the InChI treats certain groups as identical that have distinct representations in SMILES; the most common case is a doubly-bonded oxygen and an hydroxide anion attached to the same atom. The result of this is that the “canonical” labels for the individual groups will depend on their atom order. For example, different atom orders for a representation of the acetate anion will cause an interchange of the canonical labels for the hydroxyl group and the carbonyl group.

Fortunately this is not a problem in the general case, as Rule C ensures that the doubly-bonded atom is visited prior to the singly-bonded one. However, care must be taken when choosing the start atom:

Rule E: If the start atom is a negatively charged oxygen atom, start instead at any carbonyl oxygen attached to the same neighbour.

### Generation of SMILES string

#### Use of standard form

Even given a particular atom order, many different SMILES strings may be written for the same molecule. Consider ethanol, represented by the Universal SMILES string CCO. Alternative valid representations include C-C-O, C1.C12.O2, C(C(O)), [CH3]CO, and [H]C([H][H])CO. The OpenSMILES specification [21] describes a standard form for a SMILES string:

Rule F: SMILES strings should be written in the standard form described by the OpenSMILES specification.

For example, atoms in the “organic subset” (B, C, N, O, P, S, F, Cl, Br, and I) should be written as bare atomic symbols where possible (that is, CCO instead of [CH3][CH3][OH]); single bonds should be written explicitly when necessary to distinguish from an aromatic bond (that is, CCO instead of C-C-O).

#### Aromaticity

The OpenSMILES standard form referred to in Rule F indicates aromatic systems by lower case symbols rather than using the Kekulé form, for example c1ccccc1 rather than C1=CC=CC=C1 or C1C=CC=CC=1. To apply this rule it is necessary to first identify aromatic systems. Unfortunately, the OpenSMILES specification does not yet describe how to do this. Until this is described by the specification, differences between implementations of Universal SMILES will occur to the lack of a common aromatic model. Note that that this would be the case even if the Kekulé form were favoured over the aromatic form, as the choice of which Kekulé form to use relies on first identifying the associated aromatic system.

#### Cis/trans stereochemistry

The relative stereochemistry of all of the substituents around a double bond can be deduced if the relative stereochemistry of two substituents at either end is known. However, by providing explicit information on the relative stereochemistry of all substituents, the stereochemistry is clearer and errors can be more easily identified, although this comes at the expense of a longer string:

Rule G: For double bonds or allenes that exhibit a specified cis/trans stereochemistry, all of the explicit substituents should be preceded with a stereo symbol.

Example: Cl/C=C(\Br)/I not C/C=C(\Br)I

There are two choices for the direction of the slashes used for any particular cis/trans bond (or conjugated set of cis/trans bonds). To ensure a canonical representation, the same one must always be chosen:

Rule H: In any isolated or conjugated cis/trans stereochemistry system, the stereo symbol that occurs earliest should be a forward slash.

Example: Cl/C=C/I not Cl\C=C\I

The specific choice enforced by Rule H has the advantage that it minimises back slashes in the SMILES string (which in certain programming environments, e.g. the Unix command line or in Python, require a special treatment known as escaping). It also means that if a back slash *is* present in the string, then the (human) reader knows that there must be a corresponding forward slash preceding it.

Bond closures that occur at a double bond with defined stereochemistry require an additional rule as the stereo symbol can be present at one or both ends. Placing a stereo symbol at the bond closure symbol distant from the double bond should be avoided as it may lead to difficulties in interpretation:

Rule I: Cis/trans stereochemistry at a bond closure should only be indicated at the bond closure symbol attached to the double bond.

Example: C/C=C\1/NC1 not C/C=C\1/NC/1 or C/C=C1/NC/1

#### Bond closures

For every bond closure in a SMILES string, two bond closure symbols are included indicating the start and end of the bond, or the bond opening and bond closing. Once the bond is closed, the corresponding symbol is free for reuse:

Rule J: When choosing a bond closure symbol, the lowest value available should be used (where 1 is the lowest possible value). In other words, bond closure symbols should be reused once available.

When multiple bond closure symbols appear on the same atom, we need to consider the order in which the symbols should be listed:

Rule K: Where multiple bond closure symbols occur on the same atom, symbols describing bond openings are listed first, ordered by the canonical label of the corresponding neighbour atom (smallest first), followed by those describing bond closings, in the order in which the corresponding bond opening was made (i.e. the output order of the corresponding neighbour atom).

An advantage of writing out symbols for bond openings before those for bond closings is that it ensures that the same symbol does not appear twice (following the principle of reuse described by Rule J) on the same atom which, while valid according to the SMILES specification, may lead to confusion.

## Results

Support for Universal SMILES and Inchified SMILES was implemented in Open Babel version 2.3.2 [23] as an option for SMILES output (either *-xU* or *-xI*). These implementations were tested using the ChEMBL database, release 13, which contains 1,142,974 compounds as a 2D SDF file [41][42], and with a subset of the PubChem Substance database [4], the 1,041,575 compounds with SIDs from 1 to 2000000 (downloaded Aug. 7, 2012). The ChEMBL database is a highly-curated set of non-duplicate structures which have passed through a normalisation pipeline before entry. In contrast, the PubChem Substance database contains the original structures as deposited from a variety of sources; no normalisation procedure has been applied and duplicates and errors exist. For the analysis below, 11,881 (1.1%) compounds in the PubChem dataset had failures in InChI generation and so were discarded.

The following commands show how to use the *obabel* command-line program to generate Universal and Inchified SMILES strings for a structure stored in a Mol file:

C:\>**obabel figure1.mol -osmi –xU**

c1cc(/C=C/F)cc(c1)[N+](=O)[O-]

C:\>**obabel figure1.mol -osmi –xI**

c1cc(/C=C/F)cc(c1)N(=O)=O

The methods described above for generating the Universal SMILES and Inchified SMILES are such that the same SMILES string will be generated in each case in the absence of any normalisations introduced by the InChI for the Inchified SMILES. Such normalisations are quite common for the datasets studied; for only 52.5% of the ChEMBL database structures and 52.7% of the PubChem dataset are the Universal SMILES strings equal to the Inchified SMILES strings. A large proportion of the differences are due to normalisation of nitro ([N+](=O)[O-] to N(=O)=O). Normalisation of sulfoxide groups ([S+][O-] to S=O) groups also makes a small contribution. Most of the remainder is due to normalisation of tautomers. It should be noted that the normalised tautomer form may not be that which the chemist prefers; for example, all amides are converted to imidic acids.

### Shuffle test

The methods described above are recipes which, if followed, will result in a Universal SMILES or Inchified SMILES. What remains to be shown is that these methods produce a canonical identifier. The key feature of a canonical identifier is that it should be invariant to the order in which atoms are presented.

**Figure 2:**
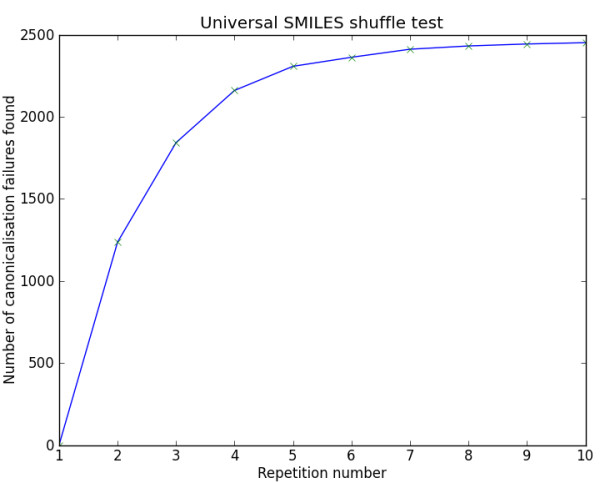
**The effect of the number of repetitions used in the shuffle test on the number of canonicalisation failures found for Universal SMILES.** This figure is based on the data from the ChEMBL database.

For each molecule in the dataset, the following procedure was used to test invariance to input atom order. Ten “anti-canonical” SMILES strings (using the *-xC* SMILES output option in Open Babel) were generated for the structure; these SMILES strings are generated by randomly assigning canonical labels to generate different output orderings. Each of these SMILES strings was then used as input to generate a Universal SMILES. If all of these were identical, the molecule was deemed to have passed the “shuffle test”. Afterwards, the whole procedure was repeated using Inchified SMILES and finally using Open Babel’s own canonical SMILES implementation. The value of ten used for the number of “anti-canonical” SMILES strings is sufficient to capture the majority of failures as shown by Figure 2.

#### ChEMBL database

When the shuffle test was applied to the ChEMBL database, there were 2,435 canonicalisation failures (0.21%) with Universal SMILES but only 141 for Inchified SMILES. This compares with 187 failures for Open Babel’s own canonical SMILES implementation. Many of these failures are due to kekulisation problems (rather than problems with the canonicalisation procedure itself), and so also affect the result for Inchified and Universal SMILES. When the failures for Open Babel’s canonical SMILES implementation are excluded, the total number of Universal SMILES failures is 2,248 while that for Inchified SMILES is zero. This value of zero is not unexpected; if the InChI successfully canonicalises the structure, the corresponding Inchified SMILES will also be canonical as a particular InChI will always yield the same Inchified SMILES.

For the Universal SMILES, a few main categories of failure could be identified. First, let us consider failures due to differences in the underlying chemical model between Open Babel and the InChI code. For example, 722 of the failures were due to disagreement on the number of tetrahedral stereocentres; typically, Open Babel identified an additional stereocentre which the InChI code correctly identified as non-stereogenic due to symmetry. Similar problems with stereogenic double bonds account for 1,105 failures. Together, these account for about 81% of the 2,248 failures.

**Figure 3:**
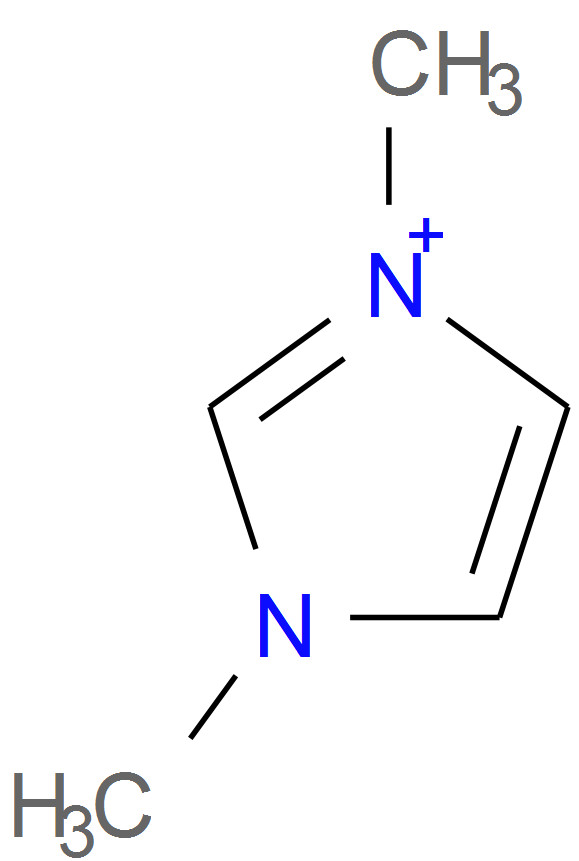
The structure of CHEMBL1229272.

Another category of failure involves handling of delocalised charges. According to the InChI FAQ [43], when computing atom numbers (during canonicalisation), bond orders and charge positions are ignored. This means that where molecular graph symmetry is broken only by charge states in a delocalised system, the InChI will regard as equivalent atoms which appear as different charge states in the SMILES string. The result of this is a non-canonical representation for the Universal SMILES string. For example, two different Universal SMILES are generated for the structure shown in Figure 3, C[n+]1ccn(C)c1 and Cn1cc[n+](C)c1, differing only in the location of the charged nitrogen.

#### PubChem dataset

When the shuffle test was applied to the 1,029,694 molecules in the PubChem dataset, there were 2,410 canonicalisation failures (0.23%) with Universal SMILES but only 163 for Inchified SMILES. Once the 232 failures for Open Babel’s canonical SMILES implementation were excluded as before, there were 2,183 failures for Universal SMILES but none for Inchified SMILES.

About 72% of the Universal SMILES failures are due to stereochemical disagreements and the majority of the remainder involve the handling of delocalised charges discussed above for ChEMBL. An additional class of failures which occurred more often in the case of the PubChem subset than with ChEMBL (56 times versus 19 times) are failures related to non-canonicalisation of isotopes. These are caused by the current lack of support for the isotopic layer as discussed at the end of the Canonical labelling section in the Methods.

**Figure 4:**
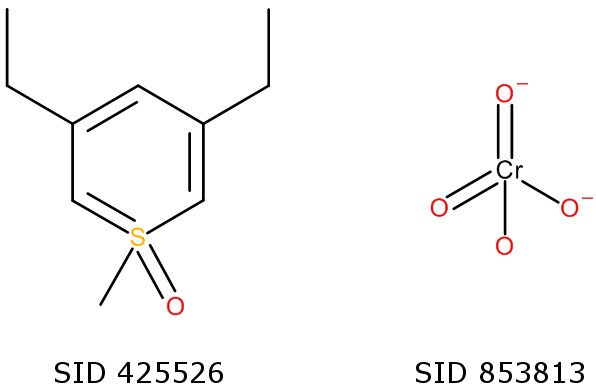
Two canonicalisation failures from the PubChem subset.

The greater diversity of the structures in PubChem yields some interesting failure cases. PubChem SID 425526 (Figure 4) is a thiopyran which Open Babel fails to canonicalise with Universal SMILES as its aromatic model differs from that of the InChI. The InChI regards the ring as aromatic and hence the molecule has two-fold graph symmetry. However, Open Babel treats the ring as a series of double bonds and so there is no graph symmetry. PubChem SID 853813 (Figure 4) is a deposited structure for the chromate ion. This structure is incorrect (one of the negative charges is on a doubly-bonded oxygen rather than a singly-bonded one) and indeed has been corrected in the PubChem Compound database. The InChI treats the charged and neutral doubly-bonded oxygens as equivalent but the graph traversal rules described in Section 3 of the Methods do not handle this situation and so their output order is not canonical.

### Duplicate test

The Universal and Inchified SMILES were used to identify duplicates in the two datasets. Such duplicates indicate either true duplicates, a shortcoming of the method or implementation, or else a normalisation of distinct structures.

#### ChEMBL database

The ChEMBL database should not contain any true duplicates. However 63 sets of duplicates (mainly pairs) in terms of the InChI were identified and removed from further consideration. Communication with the ChEMBL team revealed these to be errors in the database which had already been identified and fixed in preparation for the next release.

There were 21 duplicates according to Inchified SMILES, but after inspection none of these were due to the method itself but rather due to the underlying toolkit. Eleven duplicates involve tetrahedral stereochemistry at a nitro group which is lost by Open Babel when the InChI code normalises [N+][O-] to N=O, as Open Babel then incorrectly considers the nitrogen to be sp^2^-hybridised and not a potential stereocentre. Six occur because the Open Babel SMILES writer does not output stereo symbols for double bonds in rings of size 8 or less as these are considered to be implicitly *cis* bonds. In fact, these all appear to be true duplicates and have been reported to ChEMBL; for example, a double bond in an 8-membered ring is marked as unknown stereochemistry in one instance (CHEMBL1730955) and *cis* in the other (CHEMBL1512517), but in the original datasource (PubChem) they both link to the same structure. The remaining 4 duplicates are due to a variety of errors in Open Babel such as lack of support for chirality at an sp^3^-hybridised carbon with a hydrogen and a deuterium.

**Figure 5:**
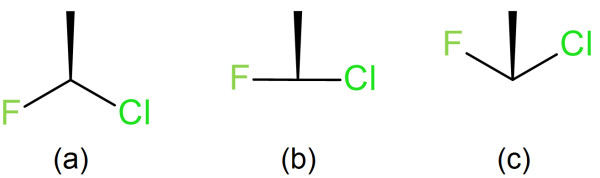
**Three arrangements of the same atoms attached to a chiral carbon that differ in the angle between the two planar bonds that includes the stereobond.** (**a**) The angle is > 180°: the hydrogen is considered to be below the plane and behind the wedge. (**b**) The angle is ~180°: some software will treat the stereochemistry as undefined depending on how close the angle is to 180°. (**c**) The angle is < 180°: the hydrogen is considered to be opposite the wedge. This is an enantiomer of (**a**).

**Figure 6:**
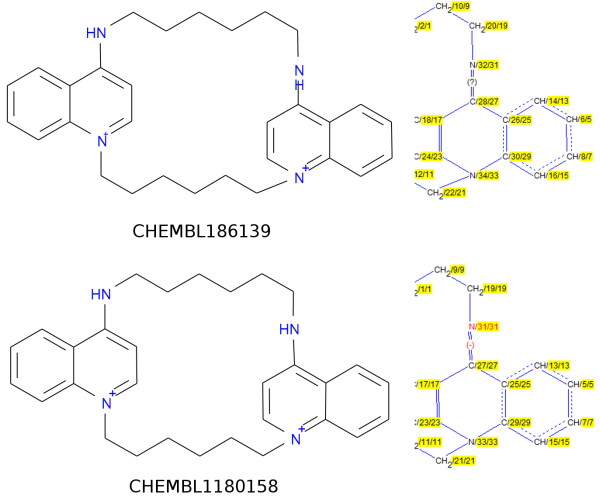
**Two entries in the ChEMBL database with seemingly identical structures but whose InChIs are distinct.** The InChIs differ in the double bond stereo layer: /b31-27+,32-28? versus /b31-27-,32-28+. The origin of the difference in InChIs is shown by the images to the right of the main structures which were generated by the *winchi* application (part of the InChI distribution).

With Universal SMILES, 29 duplicates are found which reduces to 21 after eliminating those which also occur for Inchified SMILES. The majority of these 21 appear to be true duplicates which, due to the specific way the structure is depicted in the Mol file, result in distinct InChIs. Nine of these duplicates result from the fact that the InChI software regards as undefined any tetrahedral stereocentre where one bond is implicit, there is one explicit stereo bond, and the two remaining bonds are at an angle close to 180° (Figure 5). Two other duplicates are due to poor geometry at a stereogenic double bond (two of the substituents form an angle of 60° rather than 120°) which is not accepted by the InChI software. Seven duplicates appear to be true duplicates that the InChI regards as different due to (sometimes small) variations in the angle between the two bonds of a nitrogen attached to a positively-charged pyridine (Figure 6). All of these have been reported to ChEMBL for correction. The remaining three duplicates appear to be errors in Open Babel’s handling of stereochemistry.

#### PubChem dataset

Unlike the ChEMBL database, the PubChem Substance database contains a high number of duplicates. There are 143,157 sets of duplicates in the subset of just over 1 million according to the InChI.

**Figure 7:**
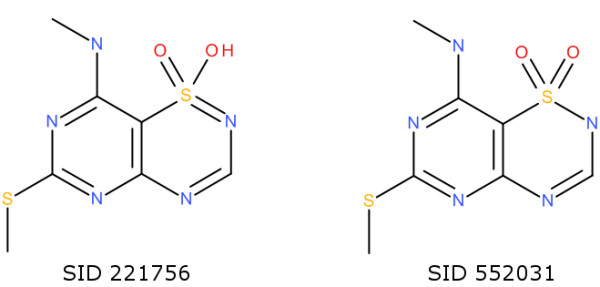
Two tautomers identified as duplicates by Inchified SMILES but not by the InChI.

**Figure 8:**
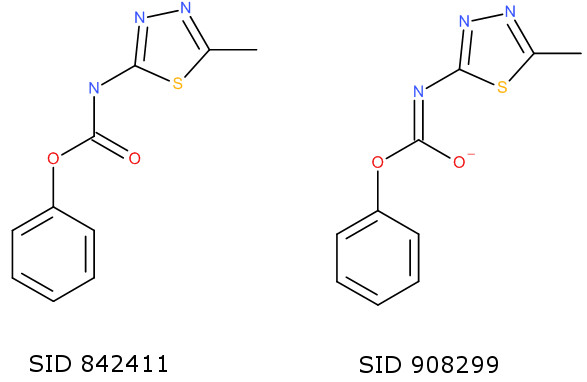
Two structures of different charge states normalised to the same neutral molecule by Inchified SMILES.

All duplicates found by the InChI were also found by the Inchified SMILES, and an additional 33 duplicates were identified. Of these, there are six cases where the InChIs are different because of a perceived double-bond stereochemistry difference across a C=N-H bond; in each case the structures link to the same CID (that is, an entry in the PubChem Compound Database). Five cases occur due to the issue with stereochemistry in 8-membered rings discussed above for the ChEMBL database; here, however, two of these cases do involve a true *trans* double bond in cyclooctene (SID 589313 and SID 594496) and so this represents a current limitation in Open Babel. Seven cases involve structures with errors which were corrected by roundtripping through the InChI. For example, the PF_6_^7-^ anion in SID 539033 and the PF_6_^5-^ anion in SID 574771 are each corrected to PF_6_^-^. Four cases involve normalisation of tautomers of heteroaromatic rings with a sulfonyl group to the same representation. For example, SIDs 221756 and 552031 (Figure 7) are tautomers (although they also have different CIDs). In three other cases the roundtrip procedure was not able to retain the original charge state and led to false positives. For example, the two structures shown in Figure 8 were normalised to the same neutral molecule. As part of the Standard InChI normalisation procedure metals are disconnected; the Inchified SMILES identified a true duplicate in the case of a ruthenium complex (SIDs 581347 and 795494) and a molecule with a coordinated chromium (SIDs 574543 and 574571), but gave to a false positive in the case of Cu=O and [Cu].O.

141,118 sets of duplicates were identified using Universal SMILES, of which all but 47 were also found by the InChI or Inchified SMILES. Of these there is one case involving the situation shown in Figure 5. 44 of the remaining cases involve a similar situation with 3D structures, where the InChI treats as undefined the stereochemistry at a tetrahedral centre as the three non-hydrogen atoms are almost in the same plane. The final two cases are further differences between the treatment of structures by Open Babel and InChI. In the case of SID 464705, Open Babel treats a molecule composed of a single hydrogen atom as a hydrogen radical, but when passed to the InChI it is interpreted as dihydrogen (this also occurs if the native InChI code is used to read the corresponding SDF file). When reading SID 823979, composed of a phosphorus atom with a double-bond to an oxygen and a single bond to an oxygen, Open Babel assumes an implicit valence of 5 for the phosphorus (indicating hypophosphorous acid) while the InChI assumes a valency of 3.

## Discussion

The implementation of Universal SMILES as part of Open Babel is able to generate canonical identifiers for 99.79% of the ChEMBL database and for 99.77% of a subset of the PubChem Substance database, a remarkable level of performance given that the InChI was never intended for such a use and that the only information taken from the InChI were the canonical labels. However, it may be possible to further improve the performance by extracting additional information. The canonicalisation failures due to stereo disagreement could be reduced by improving the ability of Open Babel to identify stereocentres. An alternative would be to try to extract information on the stereocentres from the InChI string and use that information when generating the Universal SMILES. On the other hand, there does not currently seem to be a way to resolve the failures due to delocalised charges, although future versions of the InChI could provide additional non-standard options to help here. An alternative approach would be to modify the InChI codebase itself to overcome these problems, for example as done by Cho et al. [32], but that defeats the purpose of using the InChI canonicalisation in the first place, that it exists as a standard implementation which is available within most cheminformatics toolkits.

The InChI is often used to identify and remove duplicates in chemical databases. As shown by the results of the duplicate test, the InChI software is more sensitive than Open Babel to the specific geometry used when depicting a 2D structure, and what appear to be duplicates may not be considered as such by the InChI. By comparing the duplicates found using Inchified SMILES versus Universal SMILES, some of these cases may be identified. To find the full set of problematic structures, the entire database should then be searched for additional instances (e.g. all those structures with a 180° bond angle as shown in Figure 5). These structures should then be redrawn so that the calculated InChI accurately reflects the intended structure.

The implementation of a standard SMILES representation, such as the Universal SMILES described here, will make it easy to compare SMILES strings generated by various toolkits. Some differences will be due to bugs in file format readers or writers, and their exposure will lead to improvements in quality. Other differences will be due to variations between the chemical models used by the toolkits. These variations exist between all current cheminformatics toolkits but are somewhat hidden to the casual user. Exposing these differences will encourage the development of standards for chemical models. In particular, a major obstacle in the adoption of Universal SMILES is the lack of a standard aromatic model for SMILES. The aromatic model determines which ring systems are identified as aromatic and thus affects the representation of the corresponding atoms in the SMILES string. In the absence of a standard model, each software uses different rules to determine the aromatic systems. Another issue is how to determine stereocentres, which is a difficult problem in the general case. Should each toolkit use its own code, or work together on a common standard, or should it rely on the stereocentre perception of the InChI code?

While this study has been concerned only with the SMILES format, some of the ideas described here could be applied to other chemical file formats. For example, the use of roundtripping through InChI as a normalisation step is independent of any chemical file format and could be used as a ‘business rule’ to prepare structures for entry into a database system. The canonical labels obtained from the InChI could also be used for the generation of canonical forms of other file formats, in particular for other line notations. For file formats with 2D or 3D information, a canonical ordering of the atoms makes it simple to calculate the RMSD between two docked poses (for example) as equivalent atoms occur in the same order in the two files.

## Conclusions

The normalisation and canonicalisation procedures developed by the InChI project can be used to generate a canonical form for other line notations, in particular the widely-used SMILES string. Two canonical forms were described: the Inchified SMILES incorporates the normalisations introduced by the InChI while the Universal SMILES retains the original structure. Both of these have been implemented as part of the open source toolkit Open Babel v2.3.2. For test sets of more than one million structures from the ChEMBL database and a similar-sized subset from the PubChem Substance database, no canonicalisation failures were found for Inchified SMILES, while the Universal SMILES achieved 99.8% success.

The description of these canonicalisation methods should not be considered complete, but rather as a first step in the development of an open standard for canonical SMILES. Once these methods are applied to larger and more diverse datasets, it is inevitable that further rules will be necessary to handle molecules whose structures are not currently canonicalised. Furthermore, rules for representing stereochemistries beyond tetrahedral and double-bond stereochemistry have not been considered in this work.

Although challenges remain – in particular, a description of a standard aromatic model for SMILES – the benefits to the community of a standard canonical SMILES and the ease with which it can be used to compare and contrast the chemical models used by toolkits will spur the resolution of these issues. For further discussion of the development of standards for SMILES, the interested reader is encouraged to join the OpenSMILES mailing list [21].

## Competing interests

The author declares that he has no competing interests.

## Authors’ information

The author is a developer of Open Babel, the Open Source cheminformatics toolkit, and is a member of the Blue Obelisk [44], a group which encourages interoperability and the development of standards in cheminformatics.

## Supplementary Material

Additional file 1 Python script that shows how to parse the canonical labels from an InChI with auxiliary information. Click here for file
